# Cohorts in Low- and Middle-Income Countries: From Still Photographs to Full-Length Movies

**DOI:** 10.1016/j.jadohealth.2012.09.003

**Published:** 2012-12

**Authors:** Cesar G. Victora, Fernando C. Barros

**Affiliations:** Postgraduate Program in Epidemiology, Federal University of Pelotas, Pelotas, Brazil

In recent years, the amount of information available on the health status of children and—to a lesser extent—adolescents living in low- and middle-income countries (LMICs) has been massively expanded. Population-based surveys, repeated every 5 years or so, are now available for >100 LMICs, providing information on nutritional status, health-related behaviors, morbidity, and mortality [1]. These include Demographic and Health Surveys (http://www.measuredhs.com/aboutsurveys/dhs/start.cfm) and Multiple Indicator Cluster Surveys (http://www.childinfo.org/).

However useful such cross-sectional surveys may be, they provide still photographs of the health and nutrition of young children at a given point. They do not tell us about dynamic processes such as growth, repeated disease episodes, intellectual development, or changes in health behaviors. Nor do these surveys allow linking early exposures to later health, developmental, or behavioral outcomes, an area of growing importance in life-course epidemiology since it was pioneered by David Barker approximately 30 years ago [2].

A PubMed search for birth cohort studies produces approximately 6,500 references. Of the 20 countries with the largest number of articles, only Brazil (208 references) and India (43 references) do not fall in the high-income country category. Yet, every year there are 121 million births in LMICs, and only 11 million in high-income countries [3]. Such a massive imbalance may have detrimental consequences. Results from high-income country birth cohort studies are often used to dictate global health policies, for example, warnings about the potential harmful consequences of rapid weight gain in childhood. However, results from high-income country cohorts may not be applicable to LMIC populations where poor nutrition and growth faltering are still highly prevalent, and where catch-up growth is essential not only for short-term survival but also for long-term health and human capital [4–6].

There are a number of reasons why we need more cohorts from LMICs. First, the frequency of exposures and outcomes, for example, maternal and fetal undernutrition, infectious diseases morbidity, and all-cause child mortality, is substantially higher in LMICs [3]. Second, even if their frequencies are apparently similar, exposures or health outcomes may vary substantially from one setting to another. For example, physical activity in LMICs is primarily due to commuting (on foot or bicycle), manual labor, or domestic labor, whereas in high-income countries, it is primarily due to leisure-time activities such as jogging or exercising in a gymnasium [7].

A third reason for promoting cohort studies in LMICs is that the distribution of confounding factors may be different from what is observed in high-income countries. These differences may be explored for improving causal inference from observational studies. For example, breastfeeding is usually directly associated with socioeconomic position in high-income countries (i.e., women who are richer and highly educated are most likely to breastfeed), whereas in LMICs, there is either no association or this is in the opposite direction (i.e., the poor are more likely to breastfeed) [8]. A recent comparison of the Pelotas cohort and of the UK Avon Longitudinal Study of Parents and Children (ALSPAC) cohort took advantage of the differences in confounding structures to conclude that although the positive association between breastfeeding and child intelligence was confirmed in both settings, the potential effects of breastfeeding on child obesity or blood pressure were only observed in the ALSPAC cohort, thus raising the possibility that these may have been due to residual confounding [9].

The present supplement to the *Journal of Adolescent Health* provides several additional illustrations of the value of birth cohort studies in a middle-income setting by presenting data from the 1993 Pelotas, Brazil, Birth Cohort Study—the articles focus on health, nutrition, and behaviors of 15-year-olds in 1998 [10–20]. We are fortunate to have been able to launch three population-based birth cohorts in Pelotas (in 1982, 1993, and 2004), and to continue to follow >15,000 children and adolescents up to the present day.
